# Response to the letter ‘testing the effectiveness of MyPROSLE in classifying patients with lupus nephritis’

**DOI:** 10.1093/bib/bbad454

**Published:** 2023-12-11

**Authors:** Daniel Toro-Domínguez, Jordi Martorell-Marugán, Manuel Martinez-Bueno, Raúl López-Domínguez, Elena Carnero-Montoro, Guillermo Barturen, Daniel Goldman, Michelle Petri, Pedro Carmona-Sáez, Marta E Alarcón-Riquelme

**Affiliations:** GENYO. Centre for Genomics and Oncological Research: Pfizer, University of Granada, Andalusian Regional Government, PTS Granada, Avenida de la Ilustración 114, 18016, Granada, Spain; GENYO. Centre for Genomics and Oncological Research: Pfizer, University of Granada, Andalusian Regional Government, PTS Granada, Avenida de la Ilustración 114, 18016, Granada, Spain; Fundación para la Investigación Biosanitaria de Andalucía Oriental-Alejandro Otero (FIBAO), 18012, Granada, Spain; GENYO. Centre for Genomics and Oncological Research: Pfizer, University of Granada, Andalusian Regional Government, PTS Granada, Avenida de la Ilustración 114, 18016, Granada, Spain; GENYO. Centre for Genomics and Oncological Research: Pfizer, University of Granada, Andalusian Regional Government, PTS Granada, Avenida de la Ilustración 114, 18016, Granada, Spain; Department of Statistics, University of Granada, 18071, Granada, Spain; GENYO. Centre for Genomics and Oncological Research: Pfizer, University of Granada, Andalusian Regional Government, PTS Granada, Avenida de la Ilustración 114, 18016, Granada, Spain; GENYO. Centre for Genomics and Oncological Research: Pfizer, University of Granada, Andalusian Regional Government, PTS Granada, Avenida de la Ilustración 114, 18016, Granada, Spain; Johns Hopkins University School of Medicine, Baltimore, Maryland; Johns Hopkins University School of Medicine, Baltimore, Maryland; GENYO. Centre for Genomics and Oncological Research: Pfizer, University of Granada, Andalusian Regional Government, PTS Granada, Avenida de la Ilustración 114, 18016, Granada, Spain; Department of Statistics, University of Granada, 18071, Granada, Spain; GENYO. Centre for Genomics and Oncological Research: Pfizer, University of Granada, Andalusian Regional Government, PTS Granada, Avenida de la Ilustración 114, 18016, Granada, Spain; Unit of Inflammatory Diseases, Department of Environmental Medicine, Karolinska Institute, 171 67, Solna, Sweden

**Keywords:** systemic lupus erythematosus, machine learning, transcriptomics, omics data, personalized medicine, autoimmunity

Recently, a letter to the Editor entitled ‘Testing the Effectiveness of MyPROSLE in Classifying Patients with Lupus Nephritis’ has been submitted by Leventhal et al. to Briefings in Bioinformatics. In this letter, the authors test MyPROSLE, a web application we recently introduced [1], to characterizelupus patients from the molecular point of view. Leventhal *et al*. tested the application with independent datasets reporting that the software ‘did not perform sufficiently well to consider replacement of the standard kidney biopsy as a diagnostic procedure’. In this letter, we address in detail all the concerns described by Leventhal *et al*.

First of all, we would like to thank the authors for their interest and evaluation of the web tool. Nevertheless, we want to remark that in this work we did not intend to provide software for the replacement of standard clinical diagnostic procedures, as they stated. In our manuscript, we present a scoring system to summarize the molecular portrait of each patient and machine learning models based on these features are one of the analyses used to demonstrate the utility of this scoring system. In this context, MyPROSLE was developed to apply this scoring system to gene expression datasets in order to predict clinical features based on transcriptomics data. The letter is focused on the performance of these models but, although transcriptomics profiles have emerged as a valuable resource for making new and significant discoveries in diagnosis, the integration of these profiles into clinical practice is still a distant goal. Consequently, our software was not designed to replace existing diagnostic approaches in the clinical setting but a system that can provide additional information for clinical decisions when sufficient quality RNA-Seq data is available. In the current scenario, it should be used for exploratory analysis and hypothesis generation. This concept is what we embodied in the final sentence of our original article: ‘Therefore, we set a precedent and an important advance in terms of personalized research’ (through the development of an analytical workflow) ‘oriented to a near future clinical practice within autoimmunity’.

In this letter, Leventhal *et al*. tested the capacity of MyPROSLE to predict lupus proliferative nephritis (pLN) from gene expression signatures using public blood transcriptome datasets, GSE72326 and GSE99967. These predictions were performed both, with and without healthy controls, and the agreement and Cohen’s kappa between both predictions are reported. We attempted to reproduce this analysis. Briefly, data were downloaded from National Center for Biotechnology Information (NCBI) Gene Expression Omnibus (GEO) database using the *GEOquery* R package, the expression data was transformed to a logarithmic scale and duplicated genes were merged assigning their mean expression value (following the same preprocessing guidelines as in the original article). Genes with zero or near to zero variance were filtered using *nearZeroVar* function from *caret* R package. Finally, the expression matrices were loaded into the MyPROSLE web tool to obtain the predictions of pLN for each patient (with and without healthy controls). For the dataset GSE99967 we obtained similar results for Cohen’s kappa and agreement to those obtained in Leventhal *et al*.’s letter. The dataset GSE72326 is a longitudinal study that contains several samples for each patient. Including all samples, we obtained an agreement of 84.18% and a Cohen’s kappa of 0.652, which are significantly greater than the values reported in the letter (51.7% and 0.214, respectively). To test if the discrepancy between the two results is due to a different selection of samples, we selected the first sample for each patient, obtaining an agreement of 83.9% and Cohen’s kappa of 0.656, which are very close to the ones obtained by including all the samples. The selection of the first sample is justified since it is the closest in time to the biopsies (if any) and our model was built with pLN samples validated by a recent biopsy. In fact, we used just these samples later to measure model performance. We also performed the analysis by selecting samples randomly, obtaining similar results. The code of the analysis is available athttps://github.com/GENyO-BioInformatics/MyPROSLE. Given that code and specific methodological details are not provided in the letter, we were not able to assess the reason for such discrepancy.

In our work, we analyzed the impact of incorporating healthy samples in the analysis. We analyzed the correlation between the M-scores computed with control samples and the imputed M-scores without healthy controls using a public dataset (GSE61635), obtaining a reasonable performance (Pearson correlation of 0.78, *P*-value <2.2e-16) (Figure 2B in the article [1]). We recall that the imputation of the M-scores for a new patient without providing control samples is carried out as follows: (i) the expression of the patient is centered and scaled in the same way as the expression of the samples contained in our internal database of SLE patients, (ii) Euclidean distance between the expression profile of the new patient and each patient in our database is estimated, (iii) the k most similar patients from our database to the new patient are then selected, and (iv) M-scores for the new patient are imputed as the mean of the M-scores of the k most similar patients.

For a more comprehensive assessment, we have calculated the agreement and Cohen’s kappa for 21 different datasets from different autoimmune disorders downloaded from ADEx [2] and NCBI GEO [3] (details about all used datasets are available at Github), comparing pLN predictions with and without healthy controls, finding a broad range of values (Figure 1). Furthermore, we noticed that these metrics are inversely correlated with the Euclidean distance between the target samples and the k-samples from our reference, meaning that if, for any reason, the input samples are too different from our references, the agreement will be low. We have modified the function to impute M-scores without healthy controls to limit the imputation of M-scores only to samples that surpass a max distance threshold. With this change, samples from some datasets may not be valid unless the dataset includes its own healthy controls, but it would avoid generating inaccurate results. As more patients are incorporated into the reference (from different studies and platforms), the probability of finding patients more similar to new patients in the reference will increase, thus being able to impute the M-scores more reliably without healthy controls. In fact, this problem appears when using datasets from microarray platforms that are very different from those used in our reference datasets. We have updated the MyPROSLE web tool including this improvement. Based on results from Figure 1, the threshold value was recommended as 30 by default, from which a good agreement is obtained (> ~70%).

**Figure 1:**
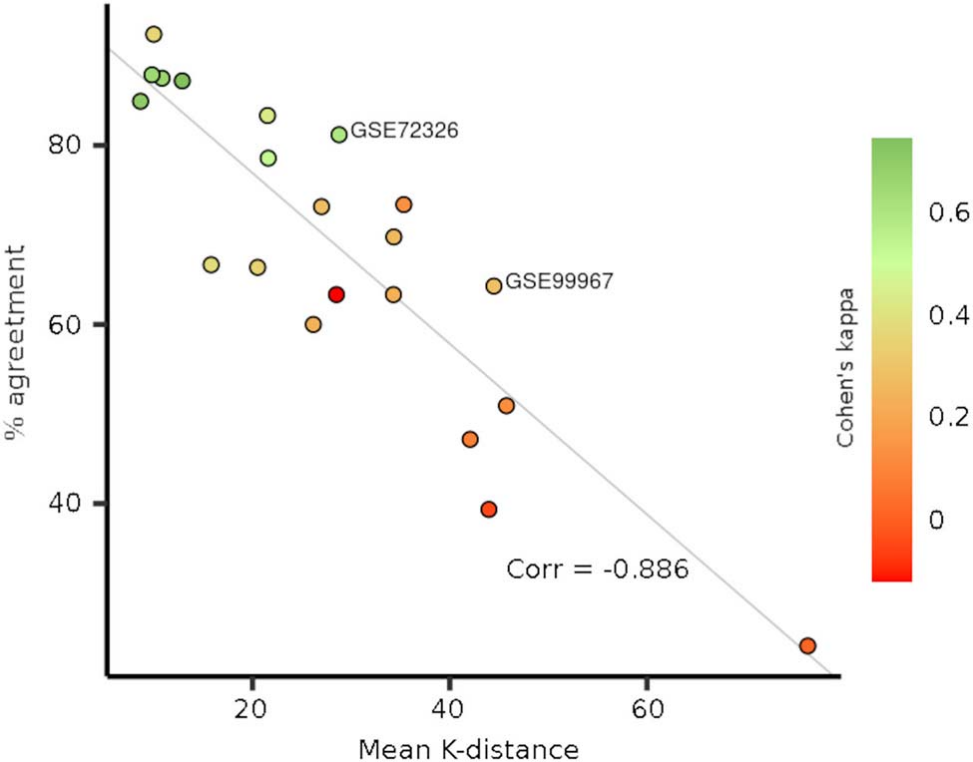
Correlation between the mean distance from k-neighbors and the percentage of agreement in predictions of different datasets. A total of 21 datasets from autoimmune disorders were downloaded from ADEx and NCBI GEO database and M-scores for each dataset were obtained from MyPROSLE web including and not including healthy samples. Agreement and Cohen’s kappa index were calculated comparing predictions for pLN with and without using healthy controls.

**Figure 2:**
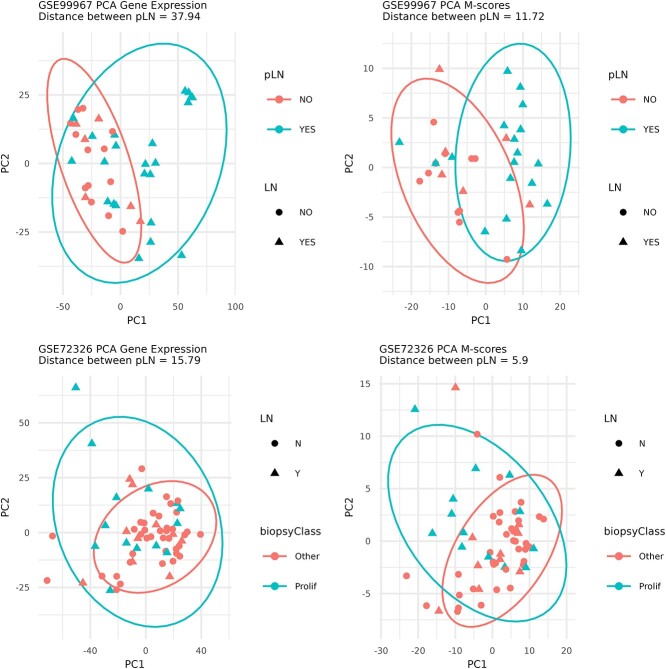
PCA of patients from the GSE99967 and GSE72326 datasets. PCAs using gene-expression data and M-scores. Euclidean distance was calculated between pLN and no-pLN samples. Only one sample for each patient was selected, for patients with a biopsy (pLN or other), we select the sample taken closest to the biopsy, for the rest of the patients, a random sample was selected.

The second major point of the letter is the prediction of pLN on the same two datasets under different conditions: selecting different samples (all nephritis cases or only pLN) and including or not including healthy samples. The model to predict pLN is validated with the GSE99967 dataset, obtaining good performance when healthy controls are included, but it is less optimal without controls. As previously commented (Figure 1) this dataset has a large Euclidean distance to our reference patients, resulting in a non-optimal imputation of the M-scores. It should be noted that calculating M-scores without using healthy controls will always be less reliable than using internal controls.

However, the model to predict pLN is not validated in the dataset GSE72326. First, we selected one sample for each patient. For patients with a biopsy, we selected the sample taken closest in time to the biopsy (the first visit for each patient). For the rest of the patients, we selected a random sample (although the results are similar if the first or a random sample for patients without pLN is selected). A balanced accuracy <0.6 was obtained (with and without healthy controls) and so, we confirmed that validation is not achieved for this dataset. We noticed that pLN samples and non-pLN samples for this dataset are less distinguishable both at the expression level and at the M-score level (Figure 2) than the GSE99967, that is, the molecular profiles of patients with and without pLN are more similar in that dataset. Analyzing the M-scores, it seems that the patients in this dataset have a higher inflammatory profile (something that usually occurs, for example, in pediatric patients), but with the available information, we can not provide a confident explanation for this.

Furthermore, we would like to remark that this model was trained with patients with active nephritis confirmed by biopsy less than a year after the sample was taken. So, it is expected that predictions will be optimal if transcriptome data is generated in patients with similar conditions. Often, samples are taken years after/before biopsies, and in these cases, it cannot be ensured that patients have active nephritis. In fact, when we trained our model, if we selected all samples regardless of the date of nephritis confirmation by biopsy, the balanced accuracy fell <0.7.

The authors also conclude that it is necessary to consider several performance metrics to evaluate the models, or that these models are influenced by the number of samples used for their training. These are obvious statements, and we would like to emphasize that we report several metrics for the models, so the users have all the information from the model to know which variables are well predicted and which are not. An alternative option is to remove non-well predicted variables, but we wanted to report all results, so we also provide information of those in which gene expression signatures did not perform well as predictors in the available data. We considered that this information was sufficient, but to avoid misunderstanding, we included a specific description of how to interpret the predictive models to highlight this fact in the web tool. Briefly, we emphasize that users should use metrics such as balanced accuracy, since this metric is more robust against possible imbalances in the data. Similarly, we advise paying close attention to the sensitivity and specificity of the different predictive models to ensure that the model predicts both classes well. For quantitative variables, users must assess the models based on the correlation and R-squared values they present. We published this tool to calculate the M-scores easily. Its usefulness is extensively demonstrated in our article because these scores are able to reflect different clinical aspects of the patients, and to predict clinical features, both for research (and not clinical, at least to date) purposes. So, more data, detailed studies and validation in clinical trials are required for the future of MyPROSLE, something we are working on.

Figure 1 of the Leventhal *et al*.’s letter contains examples of the proliferative nephritis predictions returned by MyPROSLE for false negatives and false positives. Since every machine learning classifier has an error rate, it is unfair to select specifically false positives and false negatives to be plotted. This approach could be applied to every published machine learning model and, therefore, does not provide any information about the validity of the model.

Authors also pointed out the obvious fact that the sample size is important in machine learning models and state that this information is not available in our work. However, the number of patients suffering each clinical outcome is provided in our original article. For pLN, we detailed the exact number of positive and negative cases in the text [1]. Furthermore, all the data (and the scripts) that were used in the article are contained at Github (https://github.com/GENyO-BioInformatics/MyPROSLE), so any reader can know the exact number of patients included in each model. In addition, we have now added this and additional information, such as the number of samples of each class used in each predictive model, as well as the datasets from which they came from in the table available on the web tool.

Another point in the letter refers to the influence of the missing genes for the model performance. Although we did not discuss this point in our original article, one of the advantages of M-scores is that it is calculated from the expression of several genes for each module (constructed based on co-expressed genes) and, therefore, it is a robust metric in case of missing genes. To demonstrate this, we calculated the agreement between the predictions of pLN for the whole dataset GSE99967 and afterwards we randomly selected a proportion of its genes, from 90 to 10% (Table 1). The gene selection for each proportion was repeated 10 times. As can be observed, the predictions are robust to missing genes, even when 80% of the genes are discarded (agreement = 87.1%). The code to perform this analysis is available at Github (https://github.com/GENyO-BioInformatics/MyPROSLE).

**Table 1:** **Agreement of pLN prediction removing random subsets of genes.** The table contains the percentage of randomly selected genes on GSE99967 and the mean of the agreement across 10 different iterations for each percentage

%Selected genes	90	80	70	60	50	40	30	20	10
Mean %Agreement	99.8	99.3	99.0	98.6	98.3	97.6	96.4	87.1	51.4

Finally, another aspect mentioned is that despite obtaining good precision in the prediction of pLN, such prediction was not observed for proteinuria, an associated clinical variable with active pLNs. Although they are related, we must not forget that they are two different variables, which are measured in different ways. Proteinuria is a quantitative phenotype that is categorized based on a threshold, and where the differences in magnitude within each category can be very large. For nephritis, positivity is determined by biopsy. Although there is an increase in proteinuria in patients with nephritis, not all of them have to show high values that exceed the threshold. This fact has been previously reported [4].

In dataset GSE72326 (one of the datasets used in the letter), 59.38% (38/64) of the samples with nephritis did not have proteinuria (it did not exceed the threshold of 150 mg/24 h), which means that 40% of patients with nephritis are negative for proteinuria. If we consider severe proteinuria (>300 mg/24 h), it is only present in 29.69% (19/64) of the samples with nephritis. Therefore, the fact that one prediction model works well for LN is fully compatible with the fact that another model does not work well to predict positivity for proteinuria.

In conclusion, the letter contains some reasonable concerns that have been addressed throughout this document, highlighting the discrepancy between including or not healthy controls to estimate M-scores in some datasets, which has helped us to update a part of the methodology, adding a threshold for the imputation of M-scores without the use of healthy controls, in order to obtain more reliable results. The effect of missing data has also been discussed and a more detailed explanation about how to interpret the different models contained in the web tool was required to avoid misinterpretation. Regarding the prediction of pLN, our model was confirmed using one dataset and for the second set there are several reasons that likely explain the non-validation, although we cannot know the exact reason with the information made available. We consider that Leventhal *et al*., on the one hand, have provided us with interesting contributions, but on the other hand, they have misinterpreted some aspects of our original work, such as stating that MyPROSLE is intended to replace clinical practice or the non-use of all metrics when interpreting the models. Finally, we must mention that they do not provide a sufficient level of detail for us to be able to accurately reproduce their analysis.

**Key Points**

- MyPROSLE correctly predicts proliferative nephritis for one external dataset.
- Predictive models based on M-scores are robust to missing data.
- We have incorporated a threshold on M-score imputation for samples without healthy controls to avoid using too distant samples. It is recommendable to use healthy controls if available.
- To date, MyPROSLE should be only used for research purposes, not for clinical practice.

## DATA AVAILABILITY

The code used and all data information are available at Github (https://github.com/GENyO-BioInformatics/MyPROSLE).

## FUNDING

This project has received funding from grant PID2020-119032RB-I00 supported by MCIN/AEI/10.13039/501100011033: FEDER and the Innovative Medicines Initiative 2 Joint Undertaking (JU) under grant agreement No 831434 (3TR). The JU receives support from the European Union’s Horizon 2020 research and innovation programme and EFPIA. Pedro Carmona-Sáez’s group is also funded by FEDER/Junta de Andalucía-Consejer’a de Transformación Económica, Industria, Conocimiento y Universidades (grants P20_00335 and B-CTS-40-UGR20). Daniel Toro-Domínguez is supported through the aid granted of the ‘Consejería de Transformación Económica, Industria, Conocimiento y Universidades’ (CTEICU), in the 2020 call, being co-financed by the European Union through the European Social Fund (ESF) named ‘Andalucía se mueve con Europa”, within the framework of the Andalusian ESF Operational Program 2014–2020. Guillermo Barturen is supported by a Sara Borrell grant # ISCIII CD18/00149. Jordi Martorell-Marugán is funded by Ministerio de Universidades (Spain’s Government) and the European Union – NextGenerationEU.

## Author Biographies

**Daniel Toro Domínguez** PhD in Biomedicine. He specialized in computational biology applied to the study of complex diseases, omics data analysis, machine learning and molecular clustering analysis.

**Jordi Martorell-Marugán** Postdoctoral researcher focused on the application of machine learning and other computational tools to biomedical data analysis and integration.

**Manuel Martinez-Bueno** is a researcher focused on genetic analysis applied to complex diseases.

**Raul López Domínguez** PhD in Biomedicine working in the Bioinformatics unit from GENYO.

**Elena Carnero-Montoro** is a researcher specialized in genetic epidemiology and epigenomics.

**Manuel Martinez-Bueno** is a researcher focused on genetic analysis applied to complex diseases.

**Daniel Goldman** is a professor of medicine specialized in autoimmune diseases.

**Michelle Petri** is a professor of medicine specialized in autoimmune diseases.

**Pedro Carmona-Sáez** is a professor of biostatistics with computational expertise in bioinformatics.

**Marta E. Alarcón-Riquelme** specialized in the genetics of complex diseases and autoimmunity.

## References

[1] Toro-Domínguez D, Martorell-Marugán J, Martinez-Bueno M, et al. Scoring personalized molecular portraits identify systemic lupus erythematosus subtypes and predict individualized drug responses, symptomatology and disease progression. Brief Bioinform 2022;23:bbac332. 35947992 10.1093/bib/bbac332 PMC9487588

[2] Martorell-Marugán J, López-Domínguez R, García-Moreno A, et al. A comprehensive and centralized database for exploring omics data in autoimmune diseases. BMC Bioinformatics 2021;22:343. 10.1186/s12859-021-04268-4 PMC8223391 34167460

[3] Barrett T, Wilhite SE, Ledoux P, et al. NCBI GEO: archive for functional genomics data sets--update. Nucleic Acids Res 2013;41:D991–5. 23193258 10.1093/nar/gks1193 PMC3531084

[4] De Rosa M, Rocha AS, De Rosa G, et al. Low-grade proteinuria does not exclude significant kidney injury in lupus nephritis. Kidney Int Rep 2020;5:1066–8. 32647764 10.1016/j.ekir.2020.04.005 PMC7335958

